# Automatic extraction of 12 cardiovascular concepts from German discharge letters using pre-trained language models

**DOI:** 10.1177/20552076211057662

**Published:** 2021-11-26

**Authors:** Phillip Richter-Pechanski, Nicolas A Geis, Christina Kiriakou, Dominic M Schwab, Christoph Dieterich

**Affiliations:** 1 Section of Bioinformatics and Systems Cardiology, Klaus Tschira Institute for Integrative Computational Cardiology, Heidelberg, Germany; 2 Department of Internal Medicine III, 155992 University Hospital Heidelberg, Heidelberg, Germany; 3 German Center for Cardiovascular Research (DZHK) – Partner Site Heidelberg/Mannheim, Mannheim, Germany; 4 Informatics for Life, Heidelberg, Germany

**Keywords:** Deep learning, pre-trained language models, bidirectional encoder representations from transformer, fine-tuning, medical information extraction, natural language processing

## Abstract

**Objective:**

A vast amount of medical data is still stored in unstructured text documents. We present an automated method of information extraction from German unstructured clinical routine data from the cardiology domain enabling their usage in state-of-the-art data-driven deep learning projects.

**Methods:**

We evaluated pre-trained language models to extract a set of 12 cardiovascular concepts in German discharge letters. We compared three bidirectional encoder representations from transformers pre-trained on different corpora and fine-tuned them on the task of cardiovascular concept extraction using 204 discharge letters manually annotated by cardiologists at the University Hospital Heidelberg. We compared our results with traditional machine learning methods based on a long short-term memory network and a conditional random field.

**Results:**

Our best performing model, based on publicly available German pre-trained bidirectional encoder representations from the transformer model, achieved a token-wise micro-average F1-score of 86% and outperformed the baseline by at least 6%. Moreover, this approach achieved the best trade-off between precision (positive predictive value) and recall (sensitivity).

**Conclusion:**

Our results show the applicability of state-of-the-art deep learning methods using pre-trained language models for the task of cardiovascular concept extraction using limited training data. This minimizes annotation efforts, which are currently the bottleneck of any application of data-driven deep learning projects in the clinical domain for German and many other European languages.

## Introduction

While structured reporting is an emerging field in the clinical domain, a vast amount of clinical data is still stored in unstructured text. In particular, information about patient anamnesis, cardiovascular risk factors or patient therapy is often stored as free text in discharge letters. To make these data available for research and clinical routine, we need to automatically extract relevant clinical information and store it in structured formats using methods of natural language processing (NLP).

Extracting clinical information from unstructured texts was mostly done via different pattern matching-based methods^1–3^ and statistical machine learning.^4–6^ Later, deep learning methods primarily based on recurrent neural networks (RNNs) became more and more popular,^7,8^ particularly in the field of clinical concept extraction.^9–12^ Regarding medical texts from the cardiovascular domain there had been a number of publications on English data (e.g., Small et al.,^13^ Nath et al.,^14^ Patterson et al.,^15^ and Khalifa and Meystre^16^) just a few studies focused on German texts.^17,18^ Tasks performed in the cardiovascular domain range from text classification tasks^13^ to concept and concept-value pair extraction tasks covering up to 10 concepts^19,20^ (e.g. risk factors),^16^ to a broad range of cardiovascular concepts (CCs) covering up to 80 different clinical values.^15,17,18,21^ All of the cardiovascular-related publications used either rule- and pattern-based approaches or commercial text mining tools.

Annotated clinical text corpora are rare and mostly of limited size (for details, see Supplemental Report, section-related work). This made the use of state-of-the-art supervised deep learning methods a challenge, in particular for non-English clinical texts. To overcome this obstacle transfer-learning-based methods using pre-trained language models gained recently more and more popularity in clinical information extraction (e.g. Li et al.,^22^ Beltagy et al.,^23^ Lee et al.,^24^ Si et al.,^25^ Bressem et al.,^26^ Scheible et al.^27^ and Sänger et al.^28^).

Several publications pre-trained such models, primarily based on the architecture of bidirectional encoder representations from transformers (BERTs), on English biomedical and clinical texts^23,24,29,30^ and fine-tuned them on various clinical downstream tasks (e.g. Li et al.^22^ and Si et al.^25^).

Besides general German pre-trained BERT models (https://deepset.ai/german-bert, https://github.com/dbmdz/berts, https://huggingface.co/uklfr/gottbert-base, and https://huggingface.co/deepset/gbert-large) and a model fine-tuned on medical texts collected from German internet forums and evaluated on a data set containing animal experiment reports (Non-technical Summaries of Animal Experiments)^31^ (https://huggingface.co/smanjil/German-MedBERT) there are no publicly available German pre-trained language models available.^27,32,33^ Sänger et al.^28^ used a multilingual BERT model and fine-tuned it for text classification of German animal experiment summaries. Bressem et al.^26^ pre-trained a local BERT model on chest radiographic reports and evaluated it with promising results on a text classification downstream task. To the best of our knowledge, there is no study existing, evaluating pre-training and fine-tuning language models on a concept extraction task on German discharge letters from the cardiovascular domain.

This raised the need for an in-depth evaluation of pre-training and fine-tuning BERT on a German clinical routine corpus from the cardiovascular domain to investigate the applicability of deep-learning-based NLP methods for clinical information extraction tasks.

### Objective of the study

The objective of this study was to evaluate fine-tuning of transformer-based language models based on the BERT architecture pre-trained on three different corpus types for the task of CC extraction (CCE) on limited training data. We performed our concept extraction task as a token classification task, by assigning each token to a CC or to a negative class ‘O’.

**Figure 1.**
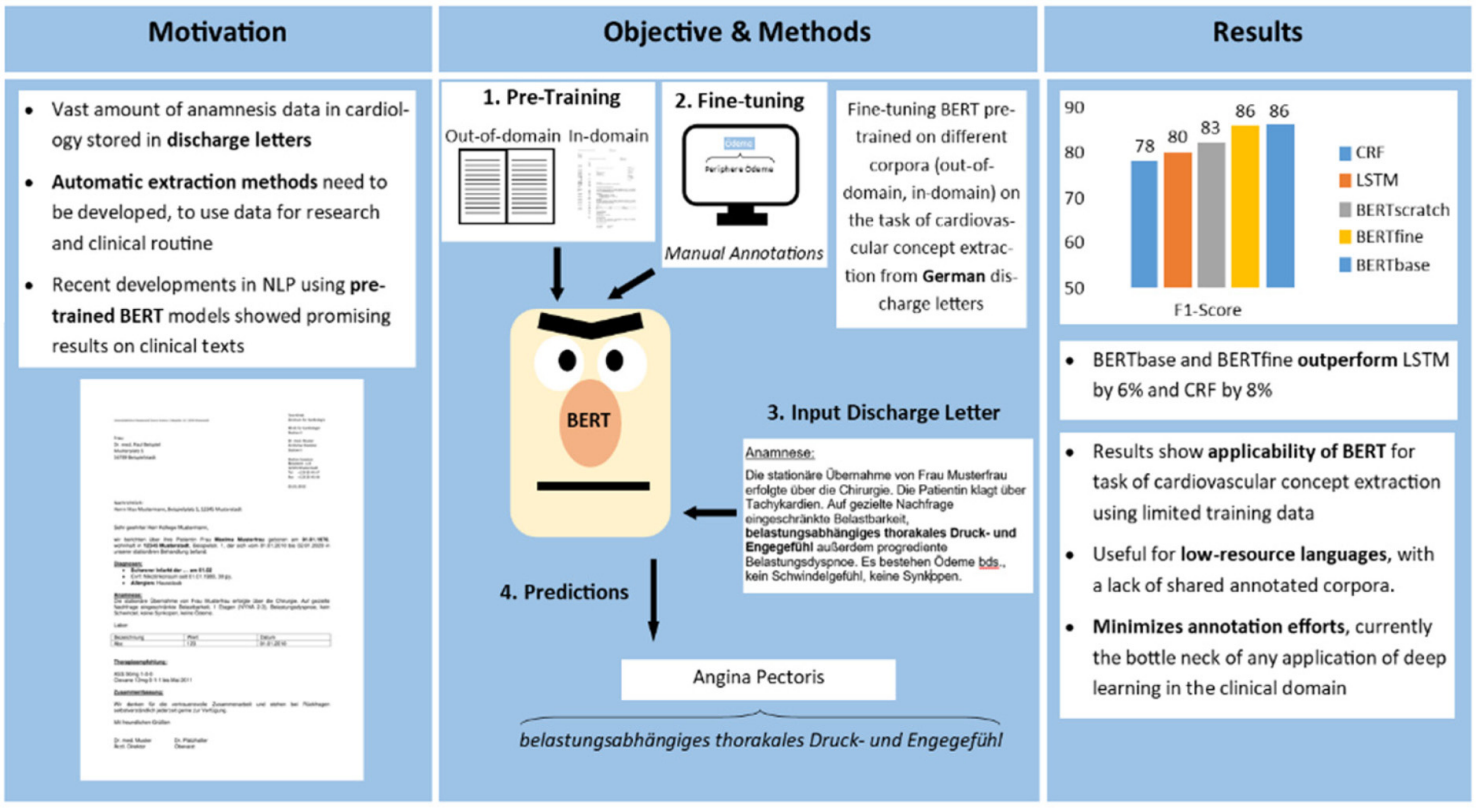
Graphical abstract: automatic extraction of 12 cardiovascular concepts (CCs) from German discharge letters using pre-trained language models.

For pre-training our BERT models, we used a corpus containing German discharge letters from the cardiology department at the Heidelberg HiGHmed (consortium of the Medical Informatics Initiative Germany) partner site. For fine-tuning BERT on our CC extraction task, we used a subset of this corpus manually annotated with 12 CCs (graphical abstract, Figure 1). This study applies existing publicly available transfer-learning methods on a novel data set in a German clinical site. This task is based on an application-driven approach. Before setting up our annotation project, we had intensive discussions with physicians to define relevant concepts for their specific domain. It was of priority to embed this task as close as possible to the clinical routine needs. In this context, this study is not aiming at benchmarking-related work results, as the selection of the concepts and the data set are use-case specific. Rather, this study is an initial investigation of the applicability of transfer-learning-based NLP methods on a local clinical use case in the cardiovascular domain.

## Methods

### Data

Our main corpus contained ∼200,000 German discharge letters in binary MS doc format covering the time period 2004 to 2020 (see Supplemental Figure S1 for an example). We applied the following pre-processing steps to the corpus:

1. Converting every discharge letter into a utf-8 encoded raw text file using the LibreOffice command-line tool (version 6.2.8). This step preserves new lines and blank lines.
2. As the task did not require the personal data of patients, we automatically de-identified each discharge letter using a deep learning approach trained on in-house data (Richter-Pechanski et al.^34^).

**Figure 2.**
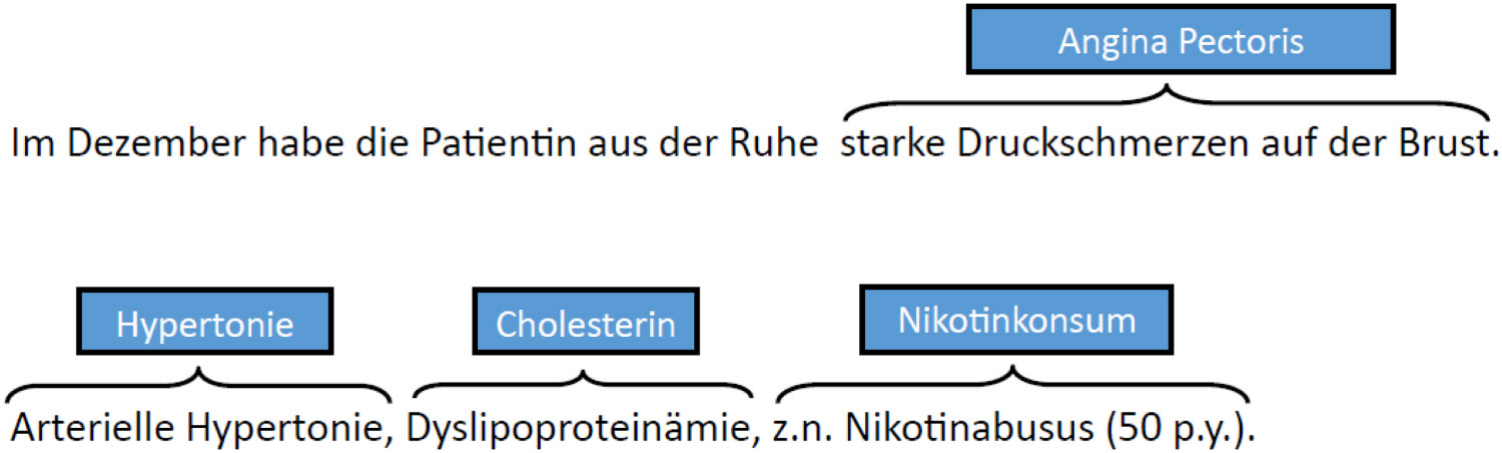
Discharge letter snippet annotated with CCs: text snippet of a discharge letter annotated with CCs. For example, the sequence ‘starke Druckschmerzen auf der Brust’ is annotated with the concept *AP*.

CC: cardiovascular concept; AP: *Angina Pectoris.*

For pre-training BERTscratch and fine-tuning BERTfine with a language model objective, we concatenated all discharge letters into a single text file. Splitting the corpus into a single token using whitespace separation, the final corpus covered ∼2 GB of text, 218,084,192 tokens in total and 667,903 unique tokens. For manual annotation and fine-tuning the models on the task of CC extraction, we selected a corpus of 204 German discharge letters (called CardioAnno) from the main corpus, covering the time period 2004 to 2016, using stratified sampling. For details on our corpus and our sampling method, see Supplemental Section 1. We tokenized the corpus using whitespace separation. We did not perform sentence splitting, due to error-prone results. Instead, we split the text by newline, thus performing a paragraph splitting. The final corpus contained 381,628 tokens in 36,355 paragraphs. Due to time limitations, we restricted the annotations to the anamnesis and the cardiovascular risk factor sections. Cardiologists involved in clinical routine carefully selected and annotated the documents with a set of 12 CCs: *Angina Pectoris* (*AP*), *Dyspnoe* (*dyspnea*), *Nykturie* (*nycturia*), *Ödeme* (*edema*), *Palpitationen* (*palpitation*), *Schwindel* (*vertigo*), *Synkope* (*syncope*), *Arterielle Hypertonie* (*Hypertonie, arterial hypertension*), *Hypercholesterinämie* (*Cholesterin, hypercholesterolemia*), *Diabetes mellitus* (*DM), Positive Familienanamnese für kardiovaskuläre Erkrankungen* ( *familial anamnesis* (FA)) and *Nikotinkonsum* (*nicotine consumption*) (Table 1). There are no nested or overlapping concepts in the data set. Figure 2 shows an annotated text snippet of a discharge letter.

**Table 1.** CCs – data analysis.

CC	ICD-10	Description	Instances	Uniqueness (%)
AP	I20	Describes a chest pain or pressure.	211	54
Dyspnoe	R06.0	Dyspnoe describes a feeling of not being able to breathe sufficiently.	215	22
Nykturie	R35	Nocturia describes the need of a patient to wake up in the night to urinate.	72	4
Ödeme	R60	Edema is the swelling of body tissue due to fluid retention.	127	28
Palpitationen	R00.2	Palpitation describes the conscious awareness of your own heartbeat.	136	17
Schwindel	H81-82	Vertigo describes the feeling of turning or swaying.	149	10
Synkope	R55	Syncope describes the sudden loss of consciousness.	168	8
Arterielle Hypertonie	I10.*	Hypertension describes the disease when the blood pressure in the arteries is persistently elevated.	175	5
Hypercholesterinämie	E78.*	This describes all appearances of cholesterols or lipids, mostly expressed as cardiovascular risk factors.	128	9
DM	E10-14	DM is a metabolic disorder characterized by high blood sugar levels.	65	8
FA	–	FA is a kind of anamnesis, which gives information about specific disease of family members.	74	11
Nikotinkonsum	F17.*	Describes a state of dependence on nicotine.	111	11

Description: Distribution of CCs in CardioAnno corpus (first column) including ICD-10 code (second column), short description (third column), number of instances (fourth column) and proportion of unique instances (fifth column).

CC: cardiovascular concept; ICD-10: International Classification of Diseases, Tenth Edition; AP: Angina Pectoris; DM: diabetes mellitus; FA: familial anamnesis.

The documents were manually annotated using a well-established iterative approach including redundant annotation, an inter-annotator agreement and guideline adaptation.^35–37^ Two annotators (assistant physicians from cardiology) achieved a token-wise inter-annotator agreement using an F1-score of 89.8%. They annotated a total of 1631 concepts. An in-depth description of the annotated corpus and the annotation process including the annotation guidelines, see Supplemental Report.

### Baseline

To compare our three pre-trained BERT models, we used two baseline classifiers, well-established methods for token classification: (i) a statistical machine learning method based on a conditional random field (CRF)^38^ and (ii) an RNN approach based on long short-term memory networks (LSTM)^39^ (we did not perform hyperparameter optimization steps for the baseline models, for more details, Supplemental Section 2).

### Pre-trained language models

Our project uses BERT, a deep learning architecture for language representation based on transformers. In contrast to traditional RNNs, transformers process input sequences in parallel. They use self-attention and positional embeddings to extract the relation between words in an input sequence and to capture its order. To apply BERT, our concept extraction task is conducted in two separate steps: (i) a pre-training step and (ii) a fine-tuning step. A randomly initialized BERT model is pre-trained on large amounts of unannotated text with two language model objectives: masked language modeling and next sentence prediction modeling. The masked language modeling objective targets predicting randomly masked (removed) tokens in an input sequence. The sentence prediction objective targets predicting, if input sentence A is followed by input sentence B.

**Figure 3.**
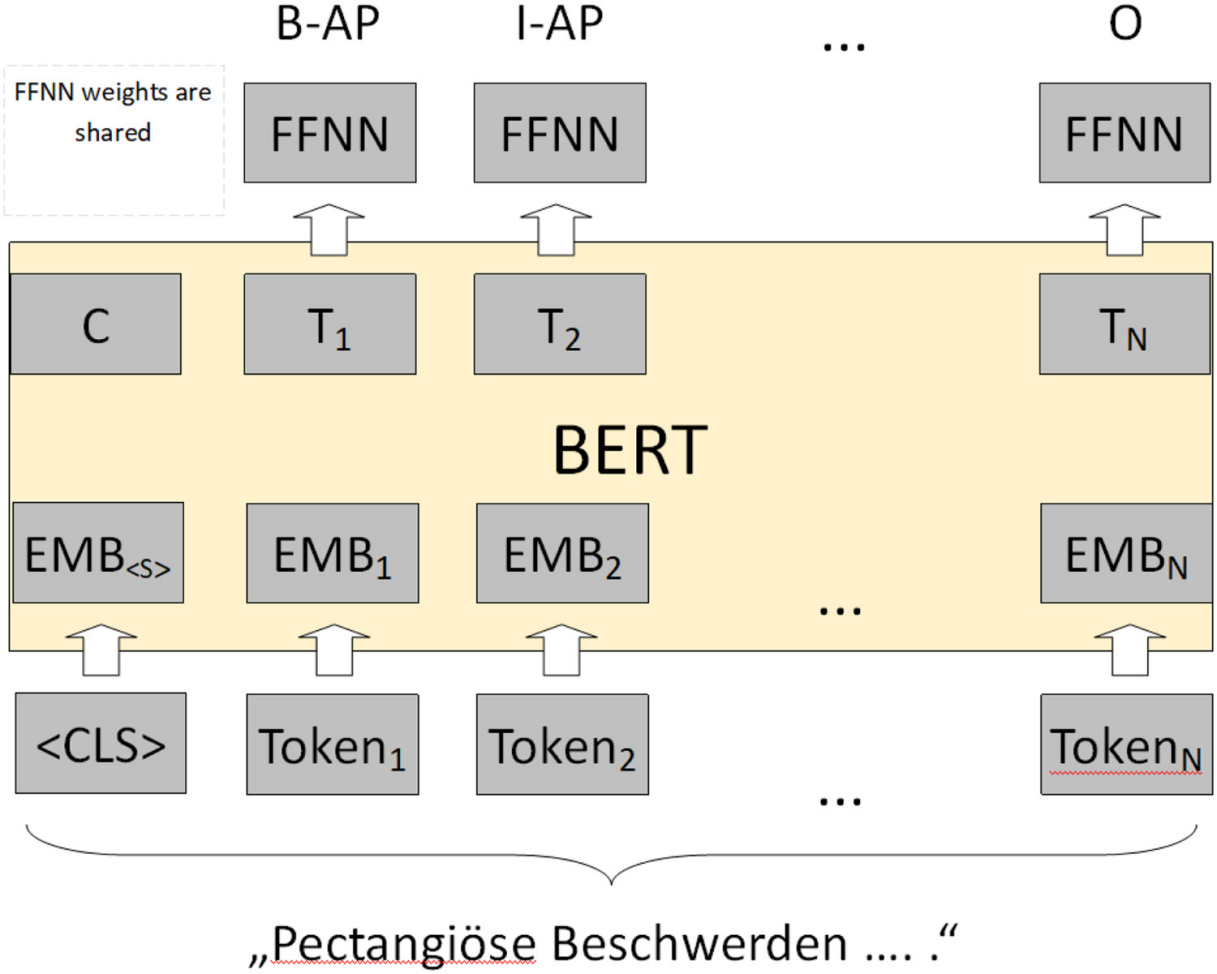
Fine-tuning BERT for cardiovascular concept extraction: input sequence ‘pectangiöse Beschwerden….’ is tokenized and embedded into a numerical representation. Each output representation T is used as input to an FFNN with a final softmax layer. For example, the token *pectangiöse* is labelled as a B-AP, the token *Beschwerden* is labelled as an I-AP sequence.^40^

BERT: bidirectional encoder representations from transformer; FFNN: feed-forward neural network; B-AP: beginning token of *Angina Pectoris*; I-AP: inner token of an *Angina Pectoris*.

After pre-training, the BERT model is fine-tuned as a supervised downstream task on annotated training data. In our task, we seek and classify phrases containing CCs. Each output vector of the BERT model is used as input to a feed-forward neural network with shared weights and a softmax layer as a final layer to classify each input token into our set of 12 concepts (Figure 3).

We evaluated three different pre-trained BERT models:

1. BERTbase: based on a publicly available German BERT model, trained on a German Wikipedia dump, an OpenLegalData dump and various news texts (https://deepset.ai/german-bert). In total, the training data size covered ∼12 GB of free text (the authors did not publish further information regarding token count in the corpus or pre-preprocessing steps).
2. BERTfine: based on BERTbase but fine-tuned with the BERT language model objectives using the complete corpus of German discharge letters at the cardiology department covering ∼2 GB of free text. We did not adapt the vocabulary of the BERTbase model.
3. BERTscratch: based on randomly initialized BERT architecture using our 2 GB corpus of discharge letters to pre-train a language model from scratch.

To perform training of BERTfine, we used the language modeling script of the HuggingFace transformer library (https://github.com/huggingface/transformers/blob/v4.0.1-release/examples/language-modeling/run_mlm.py). To perform training BERTscratch, we used the following script as a template (https://huggingface.co/blog/how-to-train). Hyperparameters: BERTfine and BERTscratch: vocab_size: 30,000; max_seq_length: 512; num_train_epochs: 3; BERTfine: per_gpu_train_batch_size: 12; BERTscratch: per_gpu_train_batch_size: 80. We used 4 ×  RTX6000 graphics processing units (GPUs) with each 24 GB video random access memory (VRAM). Training time BERTfine: ∼20 h, BERTscratch: ∼65 h. We did not perform hyperparameter optimization for pre-training and fine-tuning the BERT models under a language model objective.

## Results

To evaluate our CC classifiers, including the baseline classifiers, we used identical 4-fold cross-validation splits on the CardioAnno corpus. We calculated token-wise F1-score (the harmonic mean between precision and recall) per concept and a micro-average F1-score per classifier. We used the *HuggingFace* command-line script for token classification to fine-tune the BERT models on our concept extraction task (https://github.com/huggingface/transformers/blob/v4.0.1-release/examples/token-classification/run_ner_old.py). Training time was ∼1 h per fold using 2 ×  RTX6000 GPUs with each 24 GB VRAM. The training was performed for 30 epochs with a batch size of 16. We did not perform hyperparameter optimization steps during our experiments.

Table 2 shows the results of the baseline classifiers CRF and LSTM and the three BERT classifiers. Overall, the BERTbase and BERTfine models achieved both a similar micro-average F1-score of ∼86% and outperformed the baseline classifiers and the BERTscratch model. Per concept highest F1-scores are achieved by BERTbase (7 of 13 concepts) and BERTfine (4 of 13 concepts) and BERTscratch (3 of 13 concepts), (for more details, see Supplemental Table S1).

**Table 2.** CCE – F1-score.

CC	CRF	LSTM	BERTbase	BERTfine	BERTscratch
AP	69	73	**83**	82	78
Dyspnoe	70	72	**74**	73	70
Nykturie	96	92	**97**	91	**97**
Ödeme	57	79	91	**94**	84
Palpitation	79	74	**80**	79	77
Schwindel	87	87	95	**98**	92
Synkope	87	85	88	**89**	88
Hypertonie	89	90	**93**	87	92
Cholesterin	86	89	**92**	90	89
DM	86	90	90	**91**	**91**
FA	81	77	**82**	74	80
Nikotin	86	87	92	90	**94**
Micro average/standard deviation	78/0.83	80/1.87	**86/**1.43	**86/**1.32	83/1.98

Note: Highest values are highlighted in bold type.

Description: Mean average F1-score per concept and micro average F1-score including standard deviation of the baseline classifiers (CRF and LSTM) and the three pre-trained language models (BERTbase, BERTfine and BERTscratch) in percent. F1-score is calculated by summing up F1-scores per fold and dividing it by four.

CC: cardiovascular concept; CCE: CC extraction; CRF: conditional random field; LSTM: long short-term memory; AP: *Angina Pectoris*; DM: Diabetes mellitus; FA: *familial anamnesis*.

**Figure 4.**
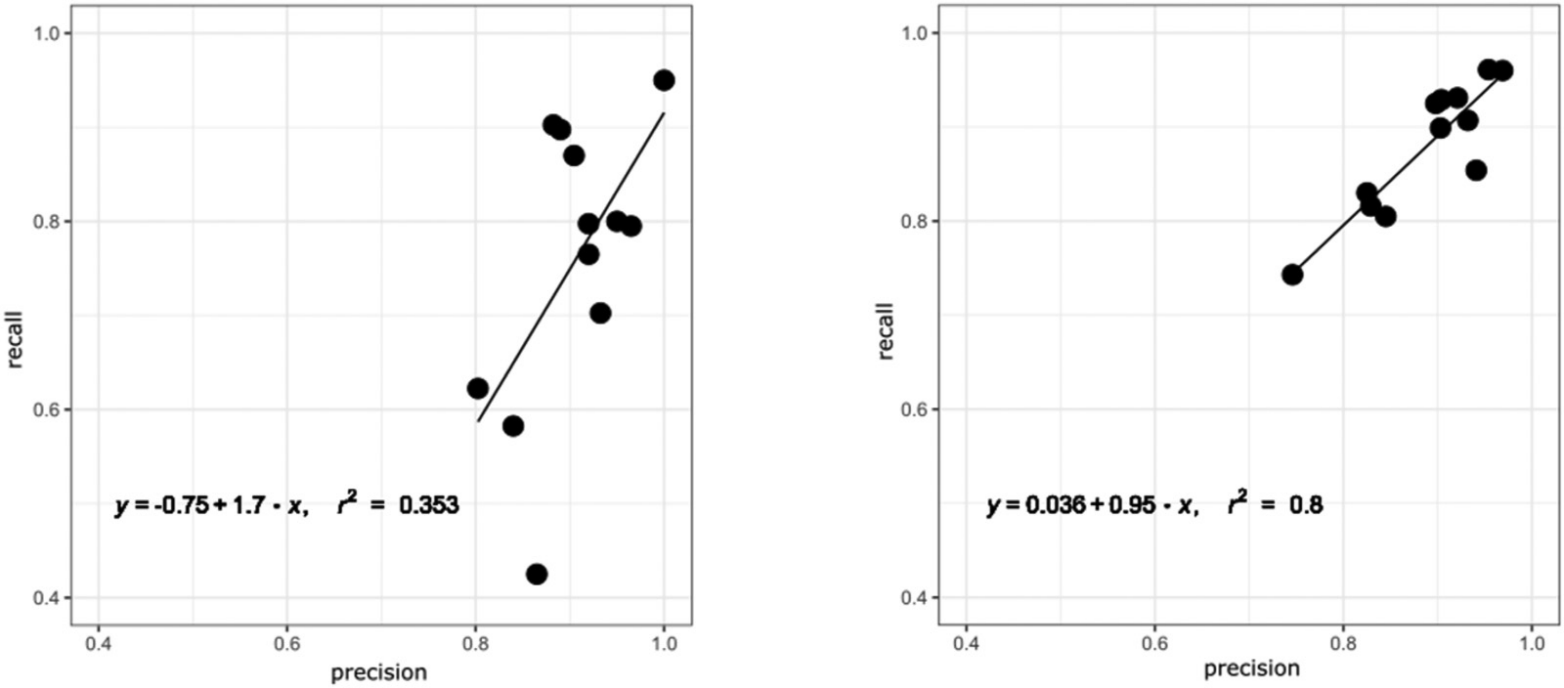
Precision/recall balance of CRF and BERTfine: balance between precision and recall per cardiovascular concept of the CRF and BERTfine model. Each data point in the scatter plots represents a CC. Defining the regression line with *y* = *b* + *ax*, an optimal result would be *r*^2^ = 1, a slope coefficient of *a* = 1 and *a* bias *b* = 0.

CRF: conditional random field; CC: cardiovascular concept.

In addition to F1-scores, we investigated the balance between precision and recall of all models per concept as visualized in Figure 4 for the worst (CRF) and the best (BERTfine) performing models. The BERTfine model achieved a slope coefficient of 0.95, with *r*^2^ of 0.8 and a bias of 0.036, while the CRF performs worse in all parameters with a bias of −0.75, a slope of 1.7 and *r*^2^ of only 0.353. In general, all BERT models achieved a better precision/recall balance than the baseline models reflecting their ability to increase recall, while keeping precision high (for detailed information see Supplemental Figure S2).

## Discussion

All three BERT models outperform the baseline models regarding micro average F1-score. However, BERTscratch achieved the lowest performance improvement to the baseline classifiers. While BERTbase and BERTfine show similar performance over most concepts, both models significantly outperform the baseline classifiers by 8% (CRF) and 6% (LSTM) regarding token-wise micro-average F1-score. We applied significant tests comparing the F1-scores of each model combination. We used approximate randomization using a threshold *p*-value <0.5 (script by Dmitry Ustalov; https://gistgithub.com/dustalov/e6c3b9d3b5b83c81ecd92976e0281d6c).^41^ The highest F1-score improvements of these models we observed for the Ödeme (>10%), AP (∼10%) and Schwindel (>8%) concepts. Significant improvements can be observed as well for the concepts: Palpitation, Nikotin, Dyspnoe and Synkope (for more details regarding significance tests on our results, see Supplemental Table S2). These concepts contain the highest number of unique instances in the corpus: AP (54%), Ödeme (28%), Dyspnoe (22%), Palpitation (17%), Nikotin (11%) and Schwindel (10%) (Table 1).

We quantitatively analysed the most common misclassifications on token level per model. We focused on false-negative classifications, as the recall was lower than precision in the majority of our evaluations. In addition, we filtered the misclassifications for the lowest-performing concepts AP, Ödeme and Dyspnea. The analysis has shown that the BERT models, especially BERTbase and BERTfine are more resistant regarding spelling mistakes. The CRF and the LSTM frequently misclassify spelling mistakes such as *Belastungsdysnpoe*, *Dypnoe*, *Blstungsdyspnoe*, etc. The same is true regarding inflected words such as *pectanginöse/m/n* or *restrosternale/m*. Explicit and frequent concept-related tokens in the data set such as *Angina*, *Dyspnoe*, *Beschwerden* or *Ödeme* are more frequently misclassified by the LSTM and CRF models. We observed frequent false-negative errors in all model predictions in the context of function words (in, der, bei, und, etc.) and punctuations (for a detailed quantitative analysis, see Supplemental Figure S3).

While the micro average recall score of the CRF (70.3) is the lowest in comparison with the LSTM (72.8) and the BERT models (81.5–85.3), the precision score of the CRF (88.5) is higher than the score of all BERT models (85.2–86.8). A similar result can be observed for the LSTM model (precision: 89.5; recall: 72.8). We observed that standard deviation of the micro average scores precision, recall and F1-score of the CRF model achieved the lowest values (0.5/0.83/0.83) suggesting the highest stability of this model, on the opposite BERTscratch had the highest standard deviation and so the lowest stability in all scores (5.2/2.5/1.98) (for details on precision and recall mean average and per fold per model, see Supplemental Table S1).

BERTfine did not achieve a significant performance gain in comparison with the general domain BERTbase model. Comparing our results with different pre-training studies, we see that the BioBERT model, fine-tuned on medical domain data sets (e.g. PubMed, PubMed + PMC) could improve performance on a biomedical entity recognition task in comparison with a general domain BERT model by increasing the data set size up to 1 billion tokens.^24^ Li et al.^22^ EhrBERT was fine-tuned on a BioBERT model on 500,000 discharge letters containing a comparable amount of token, as our data set (295 million tokens). In contrast to our observations, this model improved the results significantly in an entity normalization task on four different corpora in comparison with BioBERT. An important finding was the effect of closely related domains on the performance of BERT models. In contrast to our approach, their initial BioBERT model was already pre-trained on a closely related biomedical corpus. For future work, this should be investigated on German clinical text data: pre-training a general domain German BERT model on large amounts of publicly available German medical texts, as proposed by Borchert et al.^42^ and fine-tune this model on a local clinical corpus.

BERTscratch performed worse in comparison with BERTfine and BERTbase. Bressem et al.^26^ showed similar results on German clinical data containing radiographic reports. Performing pre-training on a data set containing ∼415 million words and 3.36 G of text their BERT model trained from scratch showed the lowest performance on a text classification task. Their best performing BERT model was initialized from the general domain German BERT model and fine-tuned on their radiographic report corpus. In contrast to BioBERT and EhrBERT, they adapted the vocabulary of their fine-tuned model to the domain-specific data set.

The effect of vocabulary adaptations needs to be investigated in future work studies. Taking the two most frequent sequences containing CCs into account (for an overview of unique token sequences per CC occurring at least two times in the corpus, see Supplemental Figure S4), we observed that BERTbase and BERTfine tokenizers split all tokens into sub-tokens, as they have not been part of the vocabulary (e.g. the sequence ‘Angina Pectoris' is split into sub-tokens *Ang, ##ina, Pe, ##ctor,* and *##is*). Regarding the 840B GloVe embeddings used for the LSTM model, six out of the 27 most frequent tokens in CC tokens are in the vocabulary. As GloVe embeddings do not use subword tokenization, out of vocabulary issues might have a more severe effect on the performance of the LSTM model. To address the out-of-vocabulary issue using traditional deep learning architectures, a valuable future work will be to compare or combine different embedding approaches, for example, FastText^43^ (using subword-level information) and character embeddings.

In the context of balance between precision and recall, the BERT models outperform the baseline classifiers in general and the CRF classifier in particular. While the CRF results per concept are skewed to a higher precision score, the BERTfine model improved recall while keeping precision high.

While our BERT approaches showed promising results, we propose a few improvements for the experimental setup. Significance tests and standard deviations of precision, recall and F1-score showed instabilities between different pieces of training/test splits for evaluation (Supplemental Table S2). Therefore, to improve the representability of the results, we need to increase the amount of training and test data. This applies particularly to the concepts with <100 instances: DM (65), Nykturie (72) and FA (74). In addition, we assume that instances such as AP, Dyspnoe and Ödeme will benefit from larger training sets, due to their high number of unique instances. To increase annotation speed, we currently apply weak supervision and active learning to support manual annotation workflows.^44^

In the annotation guidelines, we defined the constraint to restrict the physicians to exclusively annotate anamnesis and risk factor sections in discharge letters, to minimize annotation time. After discussions with the annotators followed by the manual data assessment, we could confirm that the majority of the CCs are located in these sections. Still, our manual review of the predictions showed that this constraint led to several false-positive classifications in other sections leading to ∼18% for the BERT models. To overcome this pitfall, we currently train an automatic section segmentation model for our corpus, as already done on a different corpus by Lohr et al.^45^

## Conclusion

In this study, we performed an in-depth evaluation of transfer-learning approaches using language models based on the BERT architecture pre-trained on three different corpora. We fine-tune them on German discharge letters from the cardiology domain, which are manually annotated with 12 CCs. We show that pre-trained language models outperform conventional strategies for automatic CC extraction in use-case scenarios where only limited-size training data are available. In the clinical domain annotation projects face various challenges. They rely on the knowledge of clinical experts with limited time resources. In addition data protection regulations in the European Union often prevent sharing clinical corpora with external domain experts. We are certain that local models can support the collaboration between different clinical sites by sharing deep learning architectures and foster optimization of tedious manual extraction processes in clinical daily routine, by training powerful deep learning models per clinical site, as demonstrated by our BERT CCE models. By just sharing model architectures, data protection issues can be avoided. However, sharing of a trained model may imply sharing of model vocabularies and weights, which may contain sensitive patient data.

## Supplemental Material

sj-docx-1-dhj-10.1177_20552076211057662 - Supplemental material for Automatic extraction of 12 cardiovascular concepts from German discharge letters using pre-trained language models Click here for additional data file. Supplemental material, sj-docx-1-dhj-10.1177_20552076211057662 for Automatic extraction of 12 cardiovascular concepts from German discharge letters using pre-trained language models by Phillip Richter-Pechanski, Nicolas A Geis, Christina Kiriakou, Dominic M Schwab and Christoph Dieterich in Digital Health

sj-svg-2-dhj-10.1177_20552076211057662 - Supplemental material for Automatic extraction of 12 cardiovascular concepts from German discharge letters using pre-trained language models Click here for additional data file. Supplemental material, sj-svg-2-dhj-10.1177_20552076211057662 for Automatic extraction of 12 cardiovascular concepts from German discharge letters using pre-trained language models by Phillip Richter-Pechanski, Nicolas A Geis, Christina Kiriakou, Dominic M Schwab and Christoph Dieterich in Digital Health

sj-tif-3-dhj-10.1177_20552076211057662 - Supplemental material for Automatic extraction of 12 cardiovascular concepts from German discharge letters using pre-trained language models Click here for additional data file. Supplemental material, sj-tif-3-dhj-10.1177_20552076211057662 for Automatic extraction of 12 cardiovascular concepts from German discharge letters using pre-trained language models by Phillip Richter-Pechanski, Nicolas A Geis, Christina Kiriakou, Dominic M Schwab and Christoph Dieterich in Digital Health

sj-docx-4-dhj-10.1177_20552076211057662 - Supplemental material for Automatic extraction of 12 cardiovascular concepts from German discharge letters using pre-trained language models Click here for additional data file. Supplemental material, sj-docx-4-dhj-10.1177_20552076211057662 for Automatic extraction of 12 cardiovascular concepts from German discharge letters using pre-trained language models by Phillip Richter-Pechanski, Nicolas A Geis, Christina Kiriakou, Dominic M Schwab and Christoph Dieterich in Digital Health

sj-docx-5-dhj-10.1177_20552076211057662 - Supplemental material for Automatic extraction of 12 cardiovascular concepts from German discharge letters using pre-trained language models Click here for additional data file. Supplemental material, sj-docx-5-dhj-10.1177_20552076211057662 for Automatic extraction of 12 cardiovascular concepts from German discharge letters using pre-trained language models by Phillip Richter-Pechanski, Nicolas A Geis, Christina Kiriakou, Dominic M Schwab and Christoph Dieterich in Digital Health

sj-docx-6-dhj-10.1177_20552076211057662 - Supplemental material for Automatic extraction of 12 cardiovascular concepts from German discharge letters using pre-trained language models Click here for additional data file. Supplemental material, sj-docx-6-dhj-10.1177_20552076211057662 for Automatic extraction of 12 cardiovascular concepts from German discharge letters using pre-trained language models by Phillip Richter-Pechanski, Nicolas A Geis, Christina Kiriakou, Dominic M Schwab and Christoph Dieterich in Digital Health

sj-xlsx-7-dhj-10.1177_20552076211057662 - Supplemental material for Automatic extraction of 12 cardiovascular concepts from German discharge letters using pre-trained language models Click here for additional data file. Supplemental material, sj-xlsx-7-dhj-10.1177_20552076211057662 for Automatic extraction of 12 cardiovascular concepts from German discharge letters using pre-trained language models by Phillip Richter-Pechanski, Nicolas A Geis, Christina Kiriakou, Dominic M Schwab and Christoph Dieterich in Digital Health

sj-xlsx-8-dhj-10.1177_20552076211057662 - Supplemental material for Automatic extraction of 12 cardiovascular concepts from German discharge letters using pre-trained language models Click here for additional data file. Supplemental material, sj-xlsx-8-dhj-10.1177_20552076211057662 for Automatic extraction of 12 cardiovascular concepts from German discharge letters using pre-trained language models by Phillip Richter-Pechanski, Nicolas A Geis, Christina Kiriakou, Dominic M Schwab and Christoph Dieterich in Digital Health

sj-xlsx-9-dhj-10.1177_20552076211057662 - Supplemental material for Automatic extraction of 12 cardiovascular concepts from German discharge letters using pre-trained language models Click here for additional data file. Supplemental material, sj-xlsx-9-dhj-10.1177_20552076211057662 for Automatic extraction of 12 cardiovascular concepts from German discharge letters using pre-trained language models by Phillip Richter-Pechanski, Nicolas A Geis, Christina Kiriakou, Dominic M Schwab and Christoph Dieterich in Digital Health
