# Supplementary material for: Automatic extraction of 12 cardiovascular concepts from German discharge letters using pre-trained language models
Source: Digit Health. 2021 Nov 26;7:20552076211057662. doi: 10.1177/20552076211057662 (PMC8637713; doi:10.1177/20552076211057662)
Supplement: sj-docx-1-dhj-10.1177_20552076211057662 - Supplemental material for Automatic extraction of 12 cardiovascular concepts from German discharge letters using pre-trained language models [file sj-docx-1-dhj-10.1177_20552076211057662.docx]

Supplement Figure 1

| Universitätsklinikum Musterstadt Station Sowieso \| Beispielstr. 12 \| 12345 Musterstadt |  | Test-Klinik  Zentrum für Kardiologie  Klinik für Kardiologie  Station II  Dr. med. Muster  Ärztlicher Direktor  Station II  Station Sowieso  Besipiestr. 123  12345 Musterstadt  Tel +123 23 45 67  Fax +123 23 45 66  01.01.2010 |
| --- | --- | --- |
| Frau  Dr. med. Paul Beispiel  Musterplatz 1  56789 Beispielstadt |  |  |
| Nachrichtlich:  Herrn Max Mustermann, Beispielplatz 1, 12345 Musterstadt | | |

Sehr geehrter Herr Kollege Muster,

wir berichten über Ihre Patientin Frau Maxima Musterfrau geboren am 01.01.1970, wohnhaft in 12345 Musterstadt, Beispielstr. 1, die sich vom bis in unserer sta­tio­nä­ren Be­handlung befand.

Diagnosen**:**

- Schwerer Infarkt der … am 01.02
- Cvrf: **Hyperlipidämie, Nikotinkonsum seit 01.01.1980, 30 py.**
- Allergien**:** Hausstaub

Anamnese:

Die stationäre Übernahme von Frau Musterfrau erfolgte über die Chirurgie. Die Patientin klagt über **Tachykardien**. Auf gezielte Nachfrage eingeschränkte Belastbarkeit, **belastungsabhängiges thorakales Druck- und Engegefühl** außerdem **progrediente Belastungsdyspnoe**. Es bestehen **Ödeme bds.**, **kein Schwindelgefühl**, **keine Synkopen**.

Wir danken für die vertrauensvolle Zusammenarbeit und stehen bei Rückfragen selbstverständlich jederzeit gerne zur Verfügung.

Labor:

| Bezeichnung | Wert | Datum |
| --- | --- | --- |
| Abc | 123 | 01.01.2010 |

Medikation:

ASS 50mg 1-0-0

Clexane 12mg 0-1-1 bis Mai 2011

Mit freundlichen Grüßen

Dr. med. Muster Dr. Platzhalter

Ärztl. Direktor Oberarzt

**Description:** German dummy discharge letter from cardiology domain used in CardioAnno corpus. The letters are semi-structured binary MS-doc files. Most of the letters contain at least a header with contact information, a salutation, a diagnosis section, an anamnesis, laboratory values, medication plan and a conclusion/epicrisis.
