# Supplementary material for: Automatic extraction of 12 cardiovascular concepts from German discharge letters using pre-trained language models
Source: Digit Health. 2021 Nov 26;7:20552076211057662. doi: 10.1177/20552076211057662 (PMC8637713; doi:10.1177/20552076211057662)
Supplement: sj-docx-4-dhj-10.1177_20552076211057662 - Supplemental material for Automatic extraction of 12 cardiovascular concepts from German discharge letters using pre-trained language models [file sj-docx-4-dhj-10.1177_20552076211057662.docx]

# Supplementary Figure 4: Token Sequences in CardioAnno Corpus per Cardiovascular Concept

Description: Token sequence count per cardiovascular concept occurring at least two times in the CardioAnno corpus. E.g. the token sequence *AP-Beschwerden* representing the cardiovascular concept AP occurs less than ten times in CardioAnno corpus.

### Sequence Frequency

Unique Token Sequences (> 2 Occurrences) for Concept: AP

100

80

60

40

20

Token Sequence

['Angina', 'pectoris']

0

['typische', 'pectanginöse', 'Beschwerden']

['pectanginöse', 'Beschwerden']

AP-Beschwerden

['Angina', 'Pectoris']

['Angina', 'pectoris-Beschwerden']

['pectanginösen', 'Beschwerden']

['Pectanginöse', 'Beschwerden']

['typische', 'Angina', 'pectoris']

['thorakales', 'Druckgefühl']

['Angina', 'pectoris-Symptomatik']

['pektanginöse', 'Beschwerden']

['Angina', 'pectoris', 'Beschwerden']

['thorakalen', 'Schmerzen', ',', 'die', 'in', 'den', 'linken', 'Arm', 'ausstrahlten']

['AP', 'Beschwerden']

['retrosternales', 'Druckgefühl']

['pektanginösen', 'Beschwerden']

AP-Symptomatik

['ohne', 'typische', 'pectanginöse', 'Beschwerden']

['typische', 'Angina', 'pectoris-Symptomatik']

['thorakale', 'Schmerzen']

['belastungsabhängiges', 'thorakales', 'Druck', '-', 'und', 'Engegefühl']

['persistierende', 'retrothorakale', 'Schmerzen']

['retrosternalem', 'Druck']

### Sequence Frequency

Unique Token Sequences (> 2 Occurrences) for Concept: Dyspnoe

100

80

60

40

20

0

Token Sequence

Belastungsdyspnoe

Dyspnoe

Ruhedyspnoe

['NYHA', 'II']

['progrediente', 'Belastungsdyspnoe']

['inadäquate', 'Belastungsdyspnoe']

['Dyspnoe', 'bei', 'Belastung']

['NYHA', 'I']

['belastungsabhängige', 'Dyspnoe']

['NYHA', 'III']

# Unique Token Sequences (> 2 Occurrences) for Concept: Nykturie

Sequence Frequency

120

100

80

60

40

20

0

Nykturie

Token Sequence

## Sequence Frequency

Unique Token Sequences (> 2 Occurrences) for Concept: Oedeme

100

80

60

40

20

0

Token Sequence

Ödeme

['peripheren', 'Ödeme']

['periphere', 'Ödeme']

Unterschenkelödeme

Beinödeme

['Periphere', 'Ödeme']

Knöchelödeme

Ödemen

['peripheren', 'Ödemen']

['keine', 'peripheren', 'Ödeme']

Oedeme

### Sequence Frequency

Unique Token Sequences (> 2 Occurrences) for Concept: Palpitation

100

80

60

40

20

0

Token Sequence

Palpitationen

Herzrasen

Herzrhythmusstörungen

Herzstolpern

['rezidivierend', 'auftretende', 'Episoden', 'von', 'Tachykardie', 'unterschiedlicher', 'Dauer', 'und', 'Häufigkeit']

### Sequence Frequency

Unique Token Sequences (> 2 Occurrences) for Concept: Schwindel

120

100

80

60

40

20

0

Token Sequence

Schwindel

Schwindelgefühl

['orthostatischer', 'Schwindel']

Schwindelsymptomatik

Schwindelanfälle

Schwankschwindel

['orthostatischen', 'Schwindel']

# Unique Token Sequences (> 2 Occurrences) for Concept: Synkope

Sequence Frequency

100

80

60

40

20

0

Synkopen

Synkope

synkopiert

['synkopalen', 'Ereignisse']

### Token Sequence

Sequence Frequency

Unique Token Sequences (> 2 Occurrences) for Concept: Hypertonie

120

100

80

60

40

20

0

Token Sequence

['Arterielle', 'Hypertonie']

['arterielle', 'Hypertonie']

['Arterieller', 'Hypertonus']

['art', '.', 'Hypertonie']

Hypertonus

### Sequence Frequency

Unique Token Sequences (> 2 Occurrences) for Concept: Cholesterin

120

100

80

60

40

20

0

Token Sequence

Hypercholesterinämie

Hyperlipidämie

Hyperlipoproteinämie

Dyslipoproteinämie

Dyslipidämie

# Unique Token Sequences (> 2 Occurrences) for Concept: DM

Sequence Frequency

120

100

80

60

40

20

0

['Diabetes', 'mellitus']

IDDM

['Diabetes', 'Mellitus']

Token Sequence

# Unique Token Sequences (> 2 Occurrences) for Concept: FA

Sequence Frequency

120

100

80

60

40

20

0

Familienanamnese

### Token Sequence

Sequence Frequency

Unique Token Sequences (> 2 Occurrences) for Concept: Nikotin

100

80

60

40

20

0

Token Sequence

Nikotinkonsum

Nikotinabusus

Nikotin

Ex-Nikotinabusus

Nichtraucherin

Nikotinanamnese

Ex-Nikotin

Ex-Nikotinkonsum
