# Supplementary material for: Automatic extraction of 12 cardiovascular concepts from German discharge letters using pre-trained language models
Source: Digit Health. 2021 Nov 26;7:20552076211057662. doi: 10.1177/20552076211057662 (PMC8637713; doi:10.1177/20552076211057662)
Supplement: sj-docx-5-dhj-10.1177_20552076211057662 - Supplemental material for Automatic extraction of 12 cardiovascular concepts from German discharge letters using pre-trained language models [file sj-docx-5-dhj-10.1177_20552076211057662.docx]

Supplement Report: CardioAnno – Annotating a German Clinical Corpus with Twelve Cardiovascular Concepts

**Abstract: Introduction:** While data-driven methods gained popularity in the clinical domain, a vast amount of clinical data are still stored in unstructured form. To train and evaluate these methods, unstructured data need to be carefully annotated. We present CardioAnno, a German clinical annotation project in the cardiology domain using well-established annotation methods.
**State-of-the-art:** Various published clinical annotation projects developed a fine-grained annotation process using iterative guideline adaptation, redundant annotation and an inter-annotator agreement. Up to now, there is no literature describing in-depth the annotation process itself in the cardiovascular domain. **Concept:** Due to the given time constraints in the clinical routine, we annotated a limited data set containing 203 discharge summaries from the cardiology department at the Heidelberg HiGHmed partner site. Two highly qualified cardiologists annotated these documents with twelve cardiovascular concepts using F1-score to assess inter-annotator agreement. To prove the applicability of corpora of limited size for deep learning methods, we trained a classifier for automatic concept extraction.
**Implementation:** Our final CardioAnno corpus was annotated with an inter-annotator agreement using F1-score of 89.8% token-wise, 82.5% partial entity-wise and 76.1% exact entity-wise. The classifier achieved a micro average F1-score of 80%.
**Lessons learned:** We hereby present CardioAnno, the first in-depth description of a German annotation project in the cardiology domain. We could prove, that local annotation projects for limited data sets can be performed under given time constraints of annotators working in clinical routine. The generated annotation was sufficient to train a baseline classifier for automatic cardiovascular concept extraction.

**Keywords.** Natural Language Processing, Annotation, Corpus, Machine Learning, Deep Learning, Medical Information Extraction, Concept Extraction

# Introduction

## Background

Digital health supported by state-of-the-art technologies using data-driven methods like deep learning is an emerging field in the clinical domain [1]. This makes the creation of gold standard data sets indispensable. Manually high-quality annotated text corpora need to be created to apply natural language processing (NLP) methods for automatic processing of unstructured clinical text documents. These corpora are not just valuable for training supervised machine learning algorithms, but as well for the evaluation of various types of predictive models.

Annotation projects in clinical domain face various challenges. Due to a specific medical jargon in clinical documents, annotation projects rely on profound clinical experts with limited time resources. In addition, data protection regulations in the European Union often prevent to share annotated clinical corpora with external collaborators from other scientific and clinical institutions.

## Objective and Requirements

Our objective is to show, that local annotation projects of limited size can be sufficient to train and evaluate powerful NLP models for clinical information extraction tasks. We conducted a local annotation project of limited size called CardioAnno. A corpus from cardiology domain with cardiovascular concepts (CC), carefully selected with our clinical partners at the cardiology department at the Heidelberg HiGHmed partner site was annotated. The result should meet the following requirements: the project is i. manageable under time constraints of annotators working in clinical routine, ii. the final corpus contains high-quality annotations as measured by an inter-annotator agreement (IAA), sufficient to iii. train a baseline classifier for automatic concept extraction.

# State of the art

## Related Work

There are a few publicly available corpora containing clinical routine documents in English [2]–[5] and other European languages existing [6]–[8]. Just recently the first shared German clinical corpus containing 200 discharge summaries from cancer patients had been published [9].^[[1]](#footnote-1)^ Any other existing German corpora are currently non-publicly available local projects in German clinical sites (for an overview, see [10]).

While there are several publications regarding medical information extraction on English and non-English clinical texts in general [11], just a few studies focus on the annotation process itself [12]. To standardize clinical annotation projects Wilbur et al. proposed in 2006 a guideline adaptation in combination with an IAA [13]. Three years later this has been refined to an iterative process by Roberts et al. [14]. Gurulingappa et al. used the approaches of Wilbur et al. and Roberts et al. to create a benchmark corpus for drug-related adverse effects [15]. Hahn et al. proposed a set of best practices to deal with semantically sloppy clinical entity annotation [16]. Finally Lohr et al. gave an extensive overview of their annotation project for entity recognition of five general entities (*Diagnosis, Findings, Symptoms, Anatomical Loc., Procedures*) using previously proposed iterative annotation methods on a local German clinical corpus, pointing out specific challenges, like language complexity and expert annotation disagreement, including an overview of recent annotation projects [12].

## Shortcomings

Due to the lack of large shared German corpora in general and the rare amount of published annotation projects in particular, we present an extensive description of an annotation project called CardioAnno using a local German corpus of limited size from cardiology domain. This could foster the development of such projects in Germany and support the development of joint NLP projects. To the best of our knowledge there is no specific German cardiovascular annotation project published so far. Cardiovascular discharge letters tend to contain prosaic sections especially for patient anamnesis and risk factors, where we find a lot of semantically sloppy entities [16]. To address this issue, we use well-established annotation methods and involve solely highly experienced physicians as annotators.

# Concept

**Data** We sampled a corpus of 203 German discharge letters using stratified sampling^[[2]](#footnote-2)^ from our main corpus in the Heidelberg HiGHmed partner site containing approximately 200,000 discharge letters from the cardiology department covering the time period 2004-2016 (an example discharge letter, Suppl. Figure 1). The corpus contained 381,628 token in 36,355 paragraphs. We tokenized the corpus using whitespace tokenization. For simplicity we did not do any sentence splitting, but segmented the corpus by new line characters, which defines a paragraph. Due to time restrictions, we just annotated the anamnesis and the cardiovascular risk sections. We annotated the documents with a set of twelve cardiovascular concepts, carefully selected involving cardiologists in clinical routine (*Angina Pectoris (AP), Dyspnoe, Nykturie, Ödeme, Palpitationen, Schwindel, Synkope, Arterielle Hypertonie, Hypercholesterinämie, Diabetes Mellitus (DM), Positive Familienanamnese für kardiovaskuläre Erkrankungen (FA), Nikotinkonsum*) (ICD-10 codes: Table 1, for more details, Suppl. Table 1). Figure 1 shows an annotated text snippet of a document. There are no overlapping or nested concept annotations in the data set.

**Methods** We closely followed well-established annotation methods [12]–[15], which include the usage of a guideline adaptation process by redundantly annotating documents involving an IAA in an iterative approach (Figure 2). After drafting initial annotation guidelines, a subset of documents had been sampled from the main corpus for redundant annotation. An IAA is calculated, if it is considered sufficient, the main corpus is annotated non-redundantly, otherwise the disagreements are discussed with the annotators. Based on this review the guidelines were adapted and the next iteration initiated (The guidelines can be downloaded here: https://github.com/dieterich-lab/Cardiac-Concept-Extraction/blob/master/misc/Cardio_AnnoGuides_final.pdf).

To prove applicability of the annotations for machine learning tasks we trained a deep learning model for automatic cardiovascular concept extraction using state-of-the-art long short-term memory networks (LSTM [17]) with a final CRF layer (conditional random field [18]).


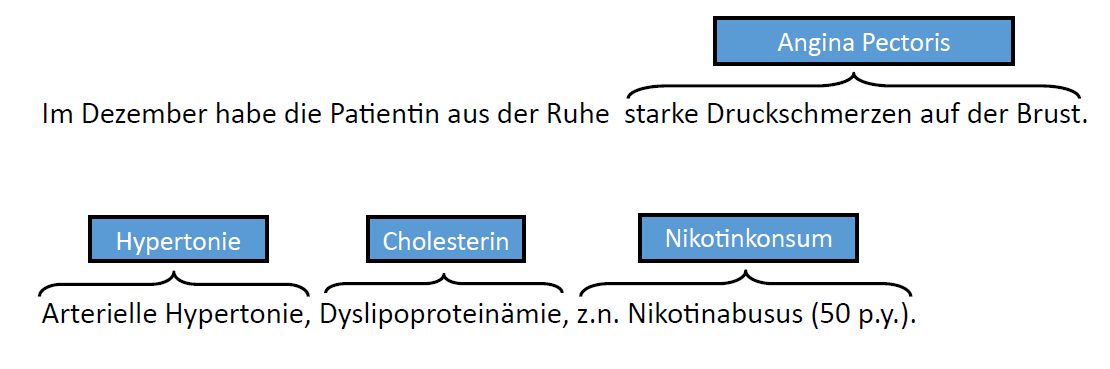


**Figure 1.** Text snippet of a discharge letter annotated with cardiovascular concepts. E.g. the sequence “*starke Druckschmerzen auf der Brust*” is annotated with the concept *AP*.


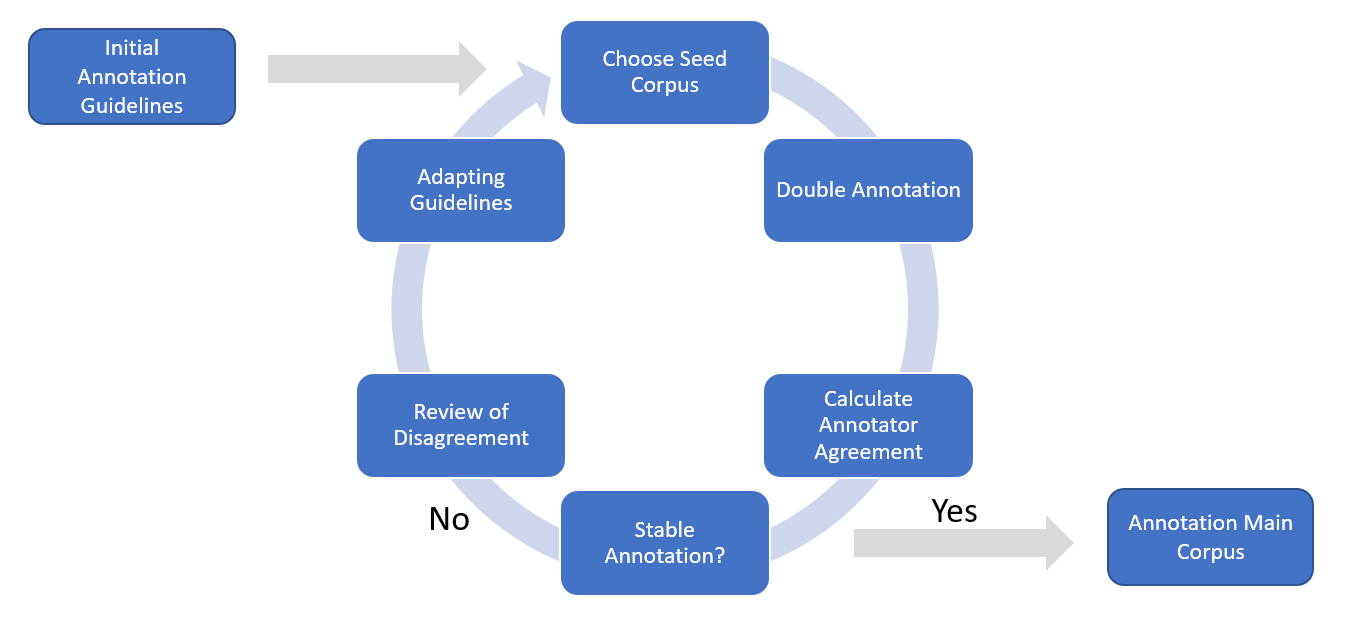
 **Figure 2.** Iterative annotation process including guideline adaptation based on redundant annotations and an IAA adapted from [14], [15].

**Participants** The project was prepared, supervised, and evaluated by an annotation master (computational linguist with four years’ experience in NLP in cardiology domain) and two annotators with clinical expertise (assistant physicians at the cardiology department with several years clinical experience in cardiology).

# Implementation

## Tools and Metrics

We used the annotation tool WebAnno optimized for span annotation, including monitoring and curation tools [19].^[[3]](#footnote-3)^ To measure the quality of the annotation an IAA is calculated. As we are performing an entity recognition task, with many token not getting annotated with a concept, we use F1-score (harmonic mean between precision and recall) as IAA score. To get a better understanding of the annotations, we applied three kinds of IAA-scores: i. an exact match IAA: both annotators completely overlap, and concept class is equal. ii. a partial match: both annotators partially or completely overlap, and concept class is equal. iii. a token-wise match: both annotators annotated the same concept class to the same token (for evaluation examples, Suppl. Table 3). The CardioAnno project lifetime including preparation, annotation and evaluation was eight months. Approximate annotation time per document was 5-10 min.

## Concept Distribution and Characteristics

Table 1 shows the distribution of annotated concepts in the corpus including the proportion of unique sequences per concept. In total 1631 concepts were annotated. The most annotations included concepts of *Dyspnoe, AP* (examples, Suppl. Figure 4) and *Arterielle Hypertonie*. The highest number of unique instances can be discovered for the concepts: *AP* (53.5%), *Ödeme* (28.3%) and *Dyspnoe* (22.3%). We analyzed the distribution of sequence length per concept (Suppl. Figure 2). Most of the concepts consist of a single token. *AP* contains the longest annotated sequences (up to 25 tokens) while *Nykturie*, *Nikotinkonsum* and *Hypercholesterinämie* primarily contain just one token per concept. *DM* and *Arterielle Hypertonie* are mostly annotated with two token (“*Diabetes Mellitus*”, “*art. Hypertonie*”), while the *Ödeme* was annotated with a maximum of seven token, but with a distribution of over 40 % each with one or two token (e.g. “*Ödeme bds.*”, “*periphere Ödeme*”). Each document contained approximately 8-9 concepts (Suppl. Figure 3).

**Table 1.** Distribution of cardiovascular concepts in CardioAnno corpus including ICD-10 code and proportion of unique instances.

| **CC** | **ICD-10** | **Instances** | **Uniqueness** |
| --- | --- | --- | --- |
| AP | I20 | 211 | 54% |
| Dyspnoe | R06.0 | 215 | 22% |
| Nykturie | R35 | 72 | 4% |
| Ödeme | R60 | 127 | 28% |
| Palpitationen | R00.2 | 136 | 17% |
| Schwindel | H81-82 | 149 | 10% |
| Synkope | R55 | 168 | 8% |
| Arterielle Hypertonie | I10.* | 175 | 5% |
| Hypercholesterinämie | E78.* | 128 | 9% |
| DM | E10-14 | 65 | 8% |
| FA | - | 74 | 11% |
| Nikotinkonsum | F17.* | 111 | 11% |

## Inter-Annotator Agreement

We performed three iterations and guideline adaptation steps including annotation review meetings to discuss disagreements^[[4]](#footnote-4)^. The annotators annotated 10-12 documents redundantly per iteration. As a quality control step, we redundantly annotated 35 documents of the main corpus (main35).

The token-wise IAA started initially at 85.9% and could be stabilized at approximately 90%. In contrast exact and partial entity-wise IAA score slightly increased in iteration 2 but decreased and stabilized approximately 5% below the initial score in the main35 split (Table 2). Looking at the IAA score per entity class (Suppl. Table 2) the concepts *Nikotinkonsum, Nykturie, Ödeme, Schwindel, FA, Hypercholesterinämie* and *AP* could be overall continuously improved during the iteration steps. Still, IAA scores for *DM* and *Dyspnoe* continuously decreased during iteration steps. Particularly risk factors like *DM* often raised disagreement, as they could appear more than once in a document. The IAA scores for *Arterielle Hypertonie*, *Palpitationen* and *Synkope* did not show a stable trend. Taking the average IAA score for the iteration steps into account, we saw a strong decrease between average score and the main35 IAA score for the semantically rich *AP* concept (Suppl. Table 2). Often annotators disagreed on descriptive token (“*typische, starke Druckschmerzen*” vs. “*starke Druckschmerzen*”, “*belastungsinduzierte pec. Beschwerden”* vs. “*pec. Beschwerden*”). *Palpitationen* and *Hypercholesterinämie* showed a slight decrease in IAA score. Disagreements for *Palpitationen* included instances, where the context was not explicit (“*Herzrythmusstörungen*”, “*Herzklopfen*”, “*Herzrasen*”). For the other concepts the IAA score could either be kept (4 concepts) or improved (5 concepts).

**Table 2.** Token-wise, partial entity-wise and exact entity-wise IAA score per iteration including main35 corpus.

| Iteration | **Token-wise** | **Partial** | **Exact** |
| --- | --- | --- | --- |
| 1 | 85.8% | 87.3% | 81.2% |
| 2 | 90.4% | 88.4% | 82.1% |
| 3 | 89.2% | 82.0% | 76.1% |
| Main 35 | 89.8% | 82.5% | 76.1% |

## Baseline Classifier

We trained a neural network based on an LSTM and a final CRF layer for automatic cardiovascular concept extraction on our manually annotated dataset. Using 4-fold cross-validation the LSTM achieved an average micro F1-score of 80.0%. (For further result details and hyperparameters see Suppl. Table 4).

# Lessons learned

Regarding our objective most requirements of our project could be met. i. Due to the intuitive annotation tool WebAnno and a well-defined annotation process, we enabled our cooperating physicians to annotate under time constraints. ii. Though several cardiovascular concepts were semantically rich and partially complex, the token-wise and partial entity-wise IAA could be kept over 80%, which is similar to previous clinical annotation projects [12]. Regular meetings and reviews of annotation disagreements assured high-quality labels. iii. We could use our annotations to train state-of-the-art deep learning algorithms for cardiovascular concept extraction on German discharge letters with sufficient performance. Currently we try to improve these results by performing experiments with recently popular pre-trained language models, like BERT, to train our concept extraction model.

Still optimizations need to be done. An error-prone restriction was to only annotate anamneses and risk factor sections. Several documents contained just implicit risk factors in diagnosis section or these sections appeared more than once in a document. This often led to confusion between the annotators. In addition, this restriction produced several misclassifications of the LSTM model, as its input was always a whole unsegmented document (document segmentation could not be reliably performed), but the LSTM found CC as well in other sections, than anamnesis or risk factor sections. Another issue was the guideline to annotate explicit grading specifications like NYHA and CCS^[[5]](#footnote-5)^ (“*CCS II*”, “*NYHA 2-3*”) only, if any other descriptive sequences for these concepts were absent in a document.

The time requirement for the annotation itself was app. 5-10 minutes per document. As the annotators were involved in clinical routine, we tried to minimize time consumption to determine review meetings using online time management tools^[[6]](#footnote-6)^. In addition, the COVID-19 pandemic led to further delay.

In a next step we plan to annotate concept negation and concept grading, such as NYHA and CCS grading for *Dyspnoe* and *AP*. This requires a larger number of annotated documents, as there is more variance to be expected (not all instances are graded, negation is not equally distributed).

Still, regarding recent experiences on clinical annotation projects, the time-limitations of clinical experts and data protection regulations, the creation of local clinical corpora to train state-of-the-art NLP models can be a promising interim solution to foster development of medical information extraction algorithms from German clinical texts. While this approach does not solve the problem of exchanging clinical texts between different clinical sites and institutions to make research results reproducible and more comprehensible, it can support the collaboration between different clinical sites by sharing deep learning architectures and foster optimizing current tedious manual extraction processes in clinical daily routine, by training powerful NLP models per clinical site, as proven by our baseline LSTM classifier.

# Conclusion

We present CardioAnno, a project to create the first annotated German clinical corpus containing discharge letters from cardiology domain with twelve cardiovascular concepts.^[[7]](#footnote-7)^ We applied well-established annotation methods and involved physicians working in daily clinical routine to assure high-quality annotations. By training a state-of-the-art deep learning model, we could prove, that already limited training data can be sufficient to train powerful classifiers for cardiovascular concept extraction to improve clinical routine procedures.

Declarations

Conflict of Interest: None

Acknowledgement: We would like to thank all members of the Dieterich Lab for their great input and insightful discussions. The work of P.R.-P. and C.D. was kindly supported by Informatics for Life funded by the Klaus Tschira Foundation and the BMBF-funded HiGHmed consortium (Medical Informatics Initiative Germany).

Authors' Contributions: P.R.-P. drafted scientific framework, prepared annotation project and supervised review meetings, curated documents, and trained the classifier. D.S. and Ch. K. annotated documents, reviewed annotations, and supported document curation. N.G. drafted clinical objective and guidelines, co-supervised review meetings and supported document curation. C.D. provided technical and scientific support and, together with all authors, prepared and reviewed the manuscript.

References

[1] U. Hahn and M. Oleynik, “Medical Information Extraction in the Age of Deep Learning,” *Yearb. Med. Inform.*, vol. 29, no. 1, (2020), pp. 208–220.

[2] M. Saeed *et al.*, “MIMIC II: a massive temporal ICU patient database to support research in intelligent patient monitoring” in *Computers in cardiology*, (2002) , pp. 641–644.

[3] A. E. W. Johnson *et al.*, “MIMIC-III, a freely accessible critical care database,” *Sci. Data*, vol. 3, no. 1, (2016), pp. 1–9.

[4] Ö. Uzuner *et al.*, “2010 i2b2/VA challenge on concepts, assertions, and relations in clinical text.” *J. Am. Med. Inform. Assoc.*, vol. 18, no. 5, (2011), pp. 552–6.

[5] W. F. Styler IV *et al.*, “Temporal annotation in the clinical domain” *Trans. Assoc. Comput. Linguist.*, vol. 2, (2014), pp. 143–154.

[6] A. Névéol *et al.*, “The QUAERO French medical corpus: A ressource for medical entity recognition and normalization” in *In proc biotextm, reykjavik*, (2014), pp. 1-7.

[7] M. Marimon *et al.*, “Automatic de-identification of medical texts in Spanish: The Meddocan track, corpus, guidelines, methods and evaluation of results” *IberLEF SEPLN*, (2019), pp. 618-638.

[8] M. Sänger *et al.*, “Classifying German Animal Experiment Summaries with Multi-lingual BERT at CLEF eHealth 2019 Task 1” (2019), pp. 1-12.

[9] M. Kittner *et al.*, “Annotation and initial evaluation of a large annotated German oncological corpus,” *JAMIA Open*, vol. 4, no. 2, (2021), pp. 1–9.

[10] F. Borchert *et al.*, “GGPONC: A Corpus of German Medical Text with Rich Metadata Based on Clinical Practice Guidelines” *arXiv Prepr. arXiv2007.06400*, (2020), pp. 1-11.

[11] I. Spasic *et al.*, “Clinical text data in machine learning: Systematic review” *JMIR Medical Informatics*, vol. 8, no. 3. (2020).

[12] C. Lohr *et al.*, “An evolutionary approach to the annotation of discharge summaries” in *Studies in Health Technology and Informatics*, vol. 270, (2020), pp. 28–32.

[13] W. J. Wilbur *et al.*, “New directions in biomedical text annotation: Definitions, guidelines and corpus construction” *BMC Bioinformatics*, vol. 7, (2006), pp. 1-10.

[14] A. Roberts *et al.*, “Building a semantically annotated corpus of clinical texts” *J. Biomed. Inform.*, vol. 42, no. 5, (2009), pp. 950–966.

[15] H. Gurulingappa *et al.*, “Development of a benchmark corpus to support the automatic extraction of drug-related adverse effects from medical case reports” *J. Biomed. Inf.*, vol. 45, no. 5, (2012), pp. 885–892.

[16] U. Hahn *et al.*, “Iterative Refinement and Quality Checking of Annotation Guidelines-How to Deal Effectively with Semantically Sloppy Named Entity Types”, LREC (2012), pp. 3881-3885.

[17] S. Hochreiter *et al.*, “Long Short-Term Memory,” *Neural Comput.*, vol. 9, no. 8, (1997), pp. 1735–1780.

[18] J. Lafferty *et al.*, “Conditional Random Fields: Probabilistic Models for Segmenting and Labeling Sequence” (2001), pp. 282-289.

[19] R. de Castilho *et al.*, “A Web-based Tool for the Integrated Annotation of Semantic and Syntactic Structures” in *Proceedings of the Workshop on LTR and Tools for Digit. Hum.*, (2016), pp. 76–84.

[20] U. Hahn *et al.*, “3000PA-Towards a national reference corpus of German clinical language” in *Studies in Health Technology and Informatics*, vol. 247, (2018), pp. 26–30.

1. Three German clinical sites announced the publication of the 3000PA corpus, but it is currently not publicly available [20]. [↑](#footnote-ref-1)
2. For details to our corpus and our sampling method, Suppl. Section 1.

   Supplement: https://github.com/dieterich-lab/Cardiac-Concept-Extraction/blob/master/README.md. [↑](#footnote-ref-2)
3. https://webanno.github.io/webanno/. [↑](#footnote-ref-3)
4. Final annotation guidelines (German) can be found here: https://github.com/dieterich-lab/Cardiac-Concept-Extraction/blob/master/misc/Cardio_AnnoGuides_final.pdf. [↑](#footnote-ref-4)
5. NYHA: <https://www.heart.org/en/health-topics/heart-failure/what-is-heart-failure/classes-of-heart-failure>, CCS: https://ccs.ca/app/uploads/2020/12/Ang_Gui_1976.pdf. [↑](#footnote-ref-5)
6. https://www.dfn.de/dienstleistungen/dfnterminplaner/. [↑](#footnote-ref-6)
7. Our task has been performed with respect to §46 Abs.2 Nr.2a (LKHG) and §13 Abs.1 Landesdatenschutzgesetz BW. In this context we had the possibility to use the data for the purpose of optimizing internal clinical procedures. [↑](#footnote-ref-7)
