# Supplementary material for: Automatic extraction of 12 cardiovascular concepts from German discharge letters using pre-trained language models
Source: Digit Health. 2021 Nov 26;7:20552076211057662. doi: 10.1177/20552076211057662 (PMC8637713; doi:10.1177/20552076211057662)
Supplement: sj-docx-6-dhj-10.1177_20552076211057662 - Supplemental material for Automatic extraction of 12 cardiovascular concepts from German discharge letters using pre-trained language models [file sj-docx-6-dhj-10.1177_20552076211057662.docx]

**Supplement Section 1**

**1.1 Corpus**

The main corpus used in this project consists of discharge letters from cardiology department as binary MS-DOC files. The corpus contains approximately 200,000 automatically de-identified discharge letters covering the time period 2004-2020 (example discharge letter, Suppl. Fig. 1). This corpus was used for pre-training our BERT models. At the time of corpus annotation though, the main corpus just contained approximately 180,000 discharge letters, covering the time period 2004-2016.

The letters (Arztbriefe) vary a lot in scope and structure between different clinical domains. They are supposed to be short and concise. Next to personal data like the name of the patient, address and birth date, most notes contain past and current diagnoses. In addition, patients' clinical history and planned clinical examinations and therapy are described. If accomplished, results of laboratory and sensor examinations are as well part of a discharge letter (details, see: https://de.wikipedia.org/wiki/Arztbrief).

Length of the discharge letters in our data set vary a lot. The letters contain between half a DIN A4 page to approximately five pages.

All documents share a basic structure. Thus, typical for clinical routine text data, they contain a semi-structure. The majority of the discharge letters contain a header containing contact information, a salutation, a clinical section and a summary. The clinical section typically contains a subset of the following subsections: diagnosis, cardiovascular risk factors, allergies, anamnese, physical examination (Körperlicher Untersuchungsbefund), laboratory data, ECG, MRI and recommended therapy/medication. The amount of text in each subsection is varying. The subsections contain free unstructured text, sometimes tables, rarely images. Occasionally subsections are titled differently, but contain similar information, e.g. therapy/medication. Often terms are abbreviated, e.g. CRF/Cardiovascular risk factors. The letters are concluded by a salutation and the names of the physicians involved.

**1.2. Sampling Method**

To obtain representative samples for annotation from our base corpus, we needed to sample a subset of discharge letters. As a probability sampling plan, to avoid too much bias, we chose stratified sampling. We performed the following steps:

- We divided our base corpus into groups of years 2004-2016. Then we randomly selected letters from each group (strata). We sampled 12 documents per strata in 2004-2012 and 24 documents per strata in 2013-2016. We chose to select twice as much documents per strata for the years 2013-2016, to keep the corpus more up-to-date.
- As our sample size is restricted to anamneses and risk factor sections of each discharge letter we obtain two constraints to our stratified sampling plan.
  - 75% of each strata we randomly selected discharge letters from the 100 largest anamneses sections (with at least 70 token) from that strata, in order to not choose letters containing too short anamnese and risk factor sections. The longest anamneses section is always included.
  - 25% of each strata we randomly selected from letters where the anamneses (with at least 45 token) contains the key words NYHA (heart failure classification, https://flexikon.doccheck.com/de/NYHA-Klassifikation) and CCS (angina pectoris classification, https://flexikon.doccheck.com/de/CCS-Klassifikation). This constraint is optional, if the key words are not available in a strata, we choose 100% from first constraint.

In total we sampled 204 discharge letters from the corpus. Following our annotation workflow for redundant annotation we split the corpus into a seed corpus (34 letters) for redundant annotation and a main corpus (170 letters). In addition the main corpus was split into a small subset containing 35 documents to annotate redundantly and a larger set of 135 documents for single annotation.
